# Knowledge and experience of Primary Education teachers regarding childhood asthma: mixed study^*^

**DOI:** 10.1590/1980-220X-REEUSP-2022-0329en

**Published:** 2023-05-22

**Authors:** Lilian Brosso, Jaqueline Brosso Zonta, Aline Fernanda Levada, Nayara Gonçalves Barbosa, Regina Aparecida Garcia Lima, Aline Cristiane Cavicchioli Okido

**Affiliations:** 1 Universidade Federal de São Carlos, Programa de Pós-Graduação em Enfermagem, São Carlos, SP, Brazil.; 2 Universidade Federal de Juiz de Fora, Faculdade de Enfermagem, Departamento de Enfermagem Materno Infantil e Saúde Pública, Juiz de Fora, MG, Brazil.; 3 Universidade de São Paulo, Escola de Enfermagem de Ribeirão Preto, Departamento de Enfermagem Materno-Infantil e Saúde Pública, Ribeirão Preto, SP, Brazil.

**Keywords:** Child Health, Asthma, Educational Personnel, Nursing, Salud Infantil, Asma, Personal Docente, Enfermería, Saúde da Criança, Asma, Pessoal de Educação, Enfermagem

## Abstract

**Objective:**

To analyze the knowledge of Primary Education teachers regarding asthma and learn about their experiences with the exacerbation of symptoms at school.

**Method:**

Sequential explanatory mixed study. In the quantitative stage, the *Newcastle Asthma Knowledge Questionnaire* and the characterization instrument were applied. Data analyzed by descriptive and inferential statistics. The production of qualitative data occurred from written statements analyzed using the deductive content analysis method.

**Results:**

Two hundred and seven teachers, mostly women (92%) and working in public schools (82%). As for knowledge, 132 (63.8%) had unsatisfactory performance. The questions with the lowest rates of correct answers were about medications used regularly and during the attacks. Teachers with higher scores had less time in the occupation (p = 0.017) and had been diagnosed with asthma (p = 0.006). In the qualitative stage, 35 teachers participated and the statements corroborated the quantitative findings, especially in relation to the knowledge gap and feeling of greater safety among asthmatic teachers.

**Conclusion:**

Teachers showed insufficient knowledge and reported fear and unpreparedness in the face of the situation.

## RESUMO

**Objetivo:**

Analisar o conhecimento de professores da Educação Básica com relação à asma e conhecer suas vivências diante da exacerbação dos sintomas na escola.

**Método:**

Estudo misto do tipo explanatório sequencial. Na etapa quantitativa, foi aplicado remotamente o *Newcastle Asthma Knowledge Questionnaire* e o instrumento de caracterização. Dados analisados por estatística descritiva e inferencial. A produção dos dados qualitativos ocorreu a partir de depoimentos escritos analisados pelo método de análise de conteúdo dedutivo.

**Resultados:**

Duzentos e sete professores, maioria do sexo feminino (92%) e atuante em escolas públicas (82%). Quanto ao conhecimento, 132 (63,8%) apresentaram desempenho insatisfatório. As questões com menores índices de acerto eram sobre medicamentos utilizados regularmente e na crise. Os professores com maiores pontuações tinham menor tempo de atuação (p = 0,017) e possuíam diagnóstico de asma (p = 0,006). Na etapa qualitativa, participaram 35 professores e os depoimentos corroboraram os achados quantitativos, sobretudo com relação à lacuna de conhecimento e sentimento de maior segurança entre os professores asmáticos.

**Conclusão:**

Os professores apresentaram conhecimento insuficiente e relataram medo e despreparo frente à situação.

## RESUMEN

**Objetivo:**

Analizar el conocimiento de docentes de Educación Básica sobre el asma y conocer sus experiencias con la agudización de los síntomas en la escuela.

**Método:**

Estudio mixto del tipo explicativo secuencial. En la etapa cuantitativa, el *Newcastle Asthma Knowledge Questionnaire* y el instrumento de caracterización fueron aplicados. Datos fueron analizados por estadística descriptiva e inferencial. La producción de datos cualitativos se basó en declaraciones escritas analizadas utilizando el método de análisis de contenido deductivo.

**Resultados:**

Doscientos siete docentes, en su mayoría mujeres el 92%) y trabajando en escuelas públicas (el 82%). En cuanto al conocimiento, 132 (el 63,8%) tuvieron desempeño insatisfactorio. Las preguntas con las tasas más bajas de respuestas correctas fueron sobre medicamentos utilizados regularmente y durante la crisis. Los docentes con puntajes más altos tenían menos tiempo de trabajo (p = 0,017) y habían sido diagnosticados con asma (p = 0,006). En la etapa cualitativa participaron 35 docentes y las declaraciones corroboraron los hallazgos cuantitativos, especialmente en relación a la brecha de conocimiento y sensación de mayor seguridad entre los docentes asmáticos.

**Conclusión:**

Los docentes tenían conocimientos insuficientes y relataron temor y falta de preparación ante la situación.

## INTRODUCTION

Asthma is a disease characterized by chronic inflammation of the airways, causing shortness of breath, wheezing, chest tightness, and cough^(1)^. It affects about 339 million people worldwide and is the most common chronic disease in childhood and adolescence^(1,2,3)^. The prevalence of childhood asthma in Latin American countries is variable, although it remains above 10% in all countries. In Brazil, the prevalence rate of asthma among children and adolescents aged between seven and 15 years living in a municipality in the southern region of the country was 16%^(4)^. Between 1996 and 2015, 5,014 children and adolescents died from asthma, the majority (68.1%) under five years of age^(3)^.

Children with asthma miss more school days when compared to children who do not have the disease, putting academic and intellectual performance at risk^(5)^. Besides the problem of school absenteeism, when without severe symptoms, children with asthma regularly attend school and spend a significant amount of time in that environment. As the symptoms of asthma exacerbation are unexpected, these children may develop acute emergencies during their time at school^(6)^. Exposure to triggers during the school day can exacerbate asthma symptoms and even be fatal^(7)^.

It is estimated that each classroom in the United States of America (USA) has an average of two to four asthmatic children, in a country where 45% of schools have a full-time nurse^(7,8)^. In Brazil, public educational institutions do not have a nursing professional, and in private institutions it is relatively common. In this context, where the constant presence of a health professional in schools is not routine, teachers begin to play an important role in providing the first aid to children and referring them to the health service, when necessary. However, teachers do not feel prepared to adequately help the child in crisis, including reports of difficulties in identifying the symptoms of asthma exacerbation^(9)^.

Faced with this problem, public programs and policies have been developed over time to strengthen prevention and health promotion actions in the school environment. Thus, the School Health Program (*PSE*) was established in 2007, through Decree No. 6.286, of December 5, 2007, as a result of a partnership between the Ministry of Health and the Ministry of Education, with the objective of improving the health of students in the public school system, establishing health education actions among students, teacher training, and early and timely identification of health problems^(10)^. Another highlight is Law No. 13.722, of October 4, 2018, which makes it mandatory for public and private primary education teaching establishments to train teachers and employees on first aid^(11)^.

In view of the above, the following research questions were established: “What is the level of knowledge of Primary Education teachers regarding asthma?”; “What sociodemographic factors are associated with the level of knowledge of these teachers?” and “How were the experiences of teachers facing a child in a situation of exacerbation of asthma symptoms in the school environment?” Therefore, this study aims at analyzing the knowledge of Primary Education teachers regarding asthma and learning about their experiences with the exacerbation of symptoms at school.

## METHOD

### Design of Study

This is a mixed method study of the sequential explanatory type, which is characterized by the collection and analysis of quantitative data and subsequent production of qualitative data to complement the knowledge about the phenomenon studied^(12)^. In this type of study, greater weight is attributed to quantitative data (QUAN) and less weight to qualitative data (Qual)^(12)^. In the present investigation, data integration took place in the results discussion stage, when the qualitative testimonies helped to explain the quantitative findings.

### Local

Due to the social restrictions imposed by the COVID-19 pandemic, data collection was carried out exclusively remotely; therefore, there was no specific location for data collection, allowing the inclusion of teachers from all regions of Brazil.

### Population and Selection Criteria

As for the study participants, non-probabilistic convenience sampling was used, with Elementary Education teachers, over 18 years of age, with at least three months of professional experience in public, private or both schools being eligible. Exclusion criteria were not established.

### Sample Size Calculation

To obtain a sample with an alpha significance level of 5% and power of 80%, the criterion adopted was *ratio of cases to IVs (Independent Variables),* which recommends 10 to 15 participants for each independent variable of the study^(13)^. The present study has ten independent variables, which will be better described later, resulting in a minimum number of 100 participants.

### Data Collection

Data collection was carried out remotely between March and May 2021. It is important to justify that at this time the Primary Education schools had already returned to their in-person activities; however, the option for the remote mode was made to meet the requirements of the COVID-19 contingency plan established by the researchers’ home institution. Initially, the research was disseminated on social media, teachers interested in participating accessed the form *online* provided by the free Google Forms platform. When entering the form, the teacher first had access to the Free and Informed Consent Form (FICF), and the filling in of the data collection instruments could be started only after the participant’s agreement.

For the production of quantitative data, an instrument of sociodemographic characterization, elaborated by the researchers, was applied, which questioned about age, sex, marital status, children, length of experience in the occupation, type of school and level of education taught, whether they had the diagnosis of asthma or if they had family members and friends with asthma and, finally, if the teacher had already experienced a situation where the student presented asthma exacerbation symptoms in the school environment.

The second instrument applied was the *Newcastle Asthma Knowledge Questionnaire* (NAKQ), developed in 1990 by Australian researchers to measure the knowledge of parents of children with asthma^(14)^. In 2016, the NAKQ was culturally adapted to Brazilian Portuguese for use among health professionals and, in 2017, among parents^(15,16)^. Among teachers, the NAKQ has been validated by Spanish scholars^(17)^. The NAKQ is composed of 31 questions, 24 with “true” or “false” answers and six open questions. The cutoff point of the instrument is ≥21 for satisfactory levels (adequate) and below 21 for unsatisfactory levels (inadequate)^(14)^.

To analyze the internal consistency of the NAKQ, Cronbach’s alpha coefficient was calculated (value equal to 0.658). This step was essential, since so far the validated version for Portuguese had not been applied among Brazilian teachers, as described above. In the Portuguese version applied among parents of children with asthma, Cronbach’s alpha coefficient was 0.71^(16)^.

To compose the qualitative empirical material, the teachers who answered positively the question of the characterization instrument “Have you ever experienced a situation where a student presented exacerbation of asthma symptoms in the school environment?” were automatically directed to another section of the form, designed to qualitatively explore their experiences. Two guiding questions were used to direct the written testimonies: Could you detail how your experience was? How did you act with the child in crisis?

### Data Analysis and Treatment

With regard to the organization of data from the instruments, they were automatically organized in a spreadsheet in Microsoft Excel and then exported to the software Statistical Analysis System for Windows, version 9.2, in which the statistical analyses were performed. In the descriptive statistical analysis, the mean, standard deviation, median, and interquartile intervals were used for discrete variables. For categorical variables, absolute and relative frequencies were used.

In this study, the knowledge score obtained from the application of the NAKQ was considered as a dependent variable, treated as a numeric variable. The independent variables were subdivided into categorical variables (sex, children, teacher’s diagnosis of asthma, experience with family members/friends with asthma, student with asthma and experience of exacerbation of asthma symptoms at school) and numeric variables (age and time of professional experience). For comparison between the dependent variable and the dichotomous categorical variables, the Mann-Whitney test was used and, for categorical variables with more than two categories, the Kruskal-Wallis test. To analyze the relationship between the knowledge score and the independent variables characterized as numerical, the Spearman Correlation Coefficient was calculated. Finally, statistically significant variables entered the univariate and multivariate linear regression model using the Stepwise Backward Wald method. The significance level adopted for the statistical tests was p < 0.05.

Data from the written testimonies were analyzed according to the deductive content analysis method, where the analysis structure is operationalized based on prior knowledge^(18)^. In the present study, the thematic categories were organized based on the two guiding questions. It is a systematized method in three stages: pre-analysis, exploration of the material and treatment of the results. In the pre-analysis stage, first a thorough reading of the testimonies was carried out and, then, exhaustive readings, to understand the empirical material produced. The material exploration stage consisted of coding the data based on their similarities and differences and subsequent grouping into thematic categories. The treatment stage of the results comprised their inference and interpretation^(18)^.

### Ethical Aspects

All regulatory norms for research with human beings described in Resolution 466/12 and Circular Letter No. 1/2021 of the National Health Council were followed. The project was approved by the Research Ethics Committee of the researchers’ home institution (information temporarily suppressed to avoid identifying the authors) under opinion No. 4.555.460, on February 24, 2021. To preserve anonymity, the participants of the qualitative stage were presented through alphanumeric coding, according to the chronological order of participation, as follows: P1, P2, and so on. People who agreed to participate in the research were instructed and signed the FICF.

## RESULTS

Two hundred and seven teachers with a mean age of 40.5 years and 14.17 years of professional experience participated in the quantitative stage. Of the total, 191 (92%) were women and 131 (63%) had children. Most worked in public schools (86%) and at Elementary School (64%). As for the diagnosis of asthma, 39 (19%) said they had the disease. With regard to the teachers’ experience of the subject, 110 (53%) stated that they had already had students with the diagnosis before and 40 (19%) had already experienced a situation of exacerbation of asthma symptoms at school.

With regard to the level of knowledge regarding asthma, 75 (36.2%) teachers scored greater than or equal to 21, corresponding to a satisfactory level of knowledge, the others (63.8%) scored below 21, presenting an unsatisfactory level of knowledge. The overall mean knowledge score was 19.34, standard deviation 3.40, minimum 11, 1st quartile 17, median 19, 3rd quartile 21, and maximum 31. The questions with the lowest rates of correct answers were related to the medications used during the attack and those used regularly to prevent the it. Only 10% of the teachers answered these questions correctly. The third question with the lowest rate of correct answers was “*Write down ways to help prevent asthma attacks during exercise,”* where 88% of the teachers made a mistake.

Table 1 presents the comparisons between the dependent variable (knowledge score) and the categorical variables of interest, with a significant difference being observed for the variables sex and diagnosis of asthma, that is, male teachers and those who claimed to be asthmatic had better performance when responding to the NAKQ.

**Table 1:** Teachers’ knowledge (n = 207) about asthma according to the categorical variables of interest – São Carlos, SP, Brazil, 2021.

Variables	n	Mean (SD)	Minimum	Q1	Median	Q3	Maximum	p value
**Sex**							
Female	191	19.18(3.32)	11	17	19	21	31.00	0.034
Male	16	21.19(3.95)	13	18.50	21	23.50	28	
**Type of Education**							
Primary	76	19.46(3.40)	11	17	20	22	30	0.072
Elementary	103	18.92(3.40)	12	17	19	21	31	
Both	28	20.54(3.19)	16	18	19.50	22.50	28	
**Type of School**							
Public	170	19.34(3.47)	11	17	19	21	31	0.893
Private	28	19.43(2.90)	16	17	19	20.50	27	
Both	9	19.00(3.81)	13	18	20	22	23	
**Children**							
Yes	131	19.24(3.31)	11	17	19	21	31	0.849
No	76	19.51(3.57)	12	17	19	21.50	30	
**Diagnosis of asthma**							
Yes	39	20.64(3.94)	12	19	21	22	31	0.010
No	168	19.04(3.20)	11	17	19	21	30	
**Asthma in family and friends**							
Yes	96	19.81(3.83)	12	17	20	22	31	0.217
No	111	18.93(2.94)	11	17	19	21	25	
**Student with asthma**							
Yes	110	19.44(3.52)	12	18	19	21	31	0.899
No	97	19.23(3.28)	11	17	20	21	27	
**Experience of asthma exacerbation at school**							
Yes	40	20.10(4.33)	12	18	19	22	31	0.418
No	167	19.16(3.13)	11	17	19	21	27	

As indicated in Table 2, the length of professional activity was negatively correlated with the knowledge score, indicating that teachers with less time of professional activity answered a greater number of questions correctly.

**Table 2:** Correlation between teachers’ knowledge (n = 207) about asthma and numerical variables of interest – São Carlos, SP, Brazil, 2021.

Variables		Age	Time in the occupation
Knowledge score	r	–0,00125	–0,17650
	p	0,9863	0,0110
	n	190	207

r = Spearman correlation coefficient; P = p-value; n = number of subjects.

Simple linear regression analysis was used in this study to assess the relationship between the independent variables and the knowledge score, and the same variables maintained a statistically significant relationship (sex, time in the occupation, and diagnosis of asthma), as shown in Table 3.

**Table 3:** Effect of independent variables on teachers’ knowledge scores (n = 207), according to a simple linear regression model – São Carlos, SP, Brazil, 2021.

Variable	Beta (SE)	p value	R^2^
Age (years)	–0.001 (0.08)	0.984	0.0000
Sex	32.89 (15.37)	**0.034**	0.0218
Time in the occupation (years)	–0.18 (0.07)	**0.011**	0.0312
Type of education	–15.08 (8.94)	0.093	0.0256
	10.34 (13.07)	0.430	
Type of school	–5.42 (12.21)	0.657	0.0011
	2.60 (20.47)	0.899	
Children	–1.63 (8.61)	0.850	0.0002
Diagnosis of asthma	27.28 (10.44)	**0.010**	0.0322
Asthma among family and friends	10.25 (8.29)	0.218	0.0074
Student with asthma	–1.06 (8.32)	0.899	0.0001
Experience of asthma exacerbation at school	8.49 (10.50)	0.420	0.0032

Beta – regression coefficient; SE: standard error of beta; R^2^ – determination coefficient.

Finally, the statistically significant variables entered the multivariate linear regression model using the Stepwise Backward Wald method and the variables “time in the occupation” and “asthma diagnosis” maintained a significant relationship with the knowledge score (p = 0.017, p = 0.006, respectively). Thus, the teachers who had the highest knowledge scores were those with the shortest time in the occupation and who claimed to have been diagnosed with asthma (Table 4).

**Table 4:** Effect of independent variables on teachers’ knowledge scores (n = 190), according to a multivariate linear regression model – São Carlos, SP, Brazil, 2021.

Variables	Categories	Beta* (SE)	p value	R^2^ partial
Diagnosis of asthma	No (ref.) Yes	– 31.03 (11.21)	**0.006**	0.0348
Time in the occupation	Continuous variable	–0.17 (0.07)	**0.017**	0.0292

Beta: estimate value or slope coefficient on the regression line; SE: standard error of beta; R^2^: determination coefficient.

**Chart 1:**
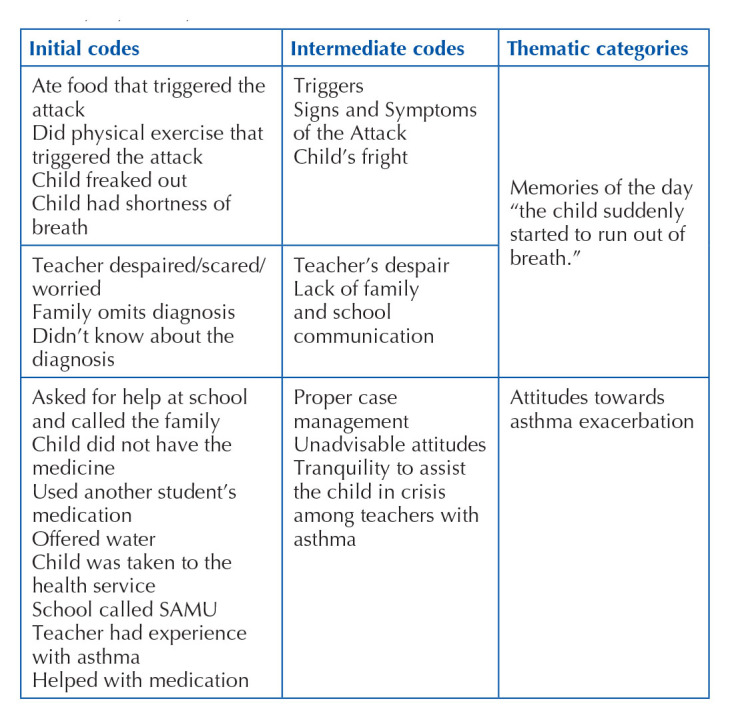
Summary of the analytical process of qualitative data – São Carlos, SP, Brazil, 2021.

Of the 207 teachers who participated in the study, 40 answered affirmatively that they had experienced a situation of asthma worsening at school, but only 35 described their experiences, with a loss of five participants. Chart 1 presents a summary of how the analytical process of the teachers’ testimonies took place until the construction of the thematic categories, which were: Memories of the day “the child suddenly started to run out of air” and “Attitudes towards asthma exacerbation”, which will be described below.

### Memories of the Day “The child Suddenly Started to Run Out of Air”

When reporting their experiences of exacerbation of asthma symptoms in the school environment, the first aspect emphasized concerns the factors that triggered the asthma attack in the child. In this direction, the practice of physical exercises was related to the episodes experienced by teachers:

*The child was participating in the physical education class and began to feel short of breath(...)* (P.4)

*The case happened after the break, where the child ran too much(...)* (P14)

*It was an episode of bronchospasm induced by exercise(...)* (P23)

The consumption of food with dye and the weather conditions were also highlighted as reasons for asthma exacerbation:

*The child ate something with dye that triggered an asthma attack(...)* (P3)

*The child had an attack on a hot, dry day* (P9).

Faced with the child’s exposure to different triggering factors, the teachers detailed in their testimonies the manifestations presented by the child in crisis. The expression used in P35’s statement accurately portrays the experience of most participants, especially since the sudden feeling of shortness of breath was the manifestation most described by teachers:

*(...) Suddenly he showed shortness of breath (...)*

Other related symptoms were also raised such as:

*(...) He coughed a lot* (P11)

*(...) He started wheezing and complaining of tiredness* (P21).

Some teachers recalled the nervousness and despair of some children, faced with the perception that their asthma symptoms were getting worse, as exemplified in the statements below:

*The child was terrified at the time of the attack.* (P5)

*The child became nervous because he could not breathe.* (P7)

The feeling of fear facing the situation was also emphasized by some teachers, especially among those who felt unprepared to help the child:

*(...) Those were horrible moments. I took her in my arms and ran (...)* (P4)

*(...) I was scared, because I didn’t know how to deal with the situation.* (P14)

According to the testimonies, another aspect that increased insecurity and fear in the face of asthma exacerbation was the lack of knowledge about the child’s diagnosis:

*(...) I didn’t know she had been diagnosed with asthma (...)* (P1)

*(...) I was not aware that she had asthma attacks.* (P26)

### Attitudes Towards Asthma Exacerbation

Although insecure and fearful with the unexpected situation, the teachers described their attitudes, to support the child under their responsibility. In this direction, some teachers reported actions aimed at reassuring the children and making them more comfortable:

*I asked her to try to stay calm, breathe slowly.* (P27).

Others requested support from members of the school team:

*I called the assistant and she took the child outside so that she could be reassured.* (P1)

*He had shortness of breath and I sent him to the coordination to call the family* (P15)

Again, the importance of prior knowledge of the child’s health condition was highlighted as a facilitating aspect. Thus, aware of the chances of the student having an asthma attack and based on a medical prescription and medication availability, the teacher acted quickly:

*The child already had an asthma report and a prescription for cases of attacks. On the day, I administered the inhaler when I noticed her shortness of breath* (P8).

The administration of inhaled medications is an indispensable action in the face of asthma exacerbation and should be started early. Therefore, it is common to keep the medicine in the backpack of asthmatic children, as exemplified below:

*In this specific case, the student carried his medication in his backpack, which was administered by me and by the school coordination.* (P28)

*The child used the inhaler he always carried in his backpack.* (P12)

*We looked for the medication in her bag (...) we shook it and applied two puffs as indicated.* (P29)

Teacher six (P6) first chose to call those responsible for the child, even with a prescription and medication available in the backpack:

*The child has severe shortness of breath, and it was necessary to call those responsible for the use of medicines.*

Teacher two (P2), on the other hand, reported his attitude of despair when not finding the medication in the backpack and not being able to contact the family:

*The student had shortness of breath at school and she didn’t have the inhaler, her mother couldn’t come and get her, I asked another student to lend her the inhaler!* (P2)

Calling the mobile pre-hospital service was also described, especially in the most severe cases:

*The child’s shortness of breath was severe. We called the mobile emergency service (SAMU), because the mother did not answer the call from the school. She was taken to the emergency room and medicated..* (P10)

*I had to call SAMU in the face of a severe crisis (...)* (P33)

Finally, familiarization with the disease, due to the fact that the teacher also had a diagnosis of asthma, was emphasized in the testimonies. According to the teachers, such personal experience brought safety and tranquility to support the child in crisis:

*As I am a chronic asthmatic, I tried to calm the child down using methods of respiratory physiotherapy and short breaths, combined with meditation until help arrived.* (P19)

*As I have asthma, I was able to help the child, making her calmer and helping her to take her medication.* (P20)

## DISCUSSION

Based on the characterization, it was observed that most of the teachers participating in the study were between 30 and 50 years old, 92% of whom were women. Studies carried out in Nigeria^(6)^, Saudi Arabia^(19)^ and United States of America^(20)^ demonstrated similar characterization. Of the 207 teachers, 132 scored below 21 on the NAKQ, indicating that most teachers had unsatisfactory levels of knowledge about asthma; however, the overall mean of knowledge in the present study was 19.34, a higher score when compared to the investigation carried out in a coastal city in Spain, where the mean was 15.7 points after applying the NAKQ to 537 teachers^(17)^.

The low performance of the teachers in this study with regard to knowledge about asthma management at school validates a result already expected by the researchers, especially since the Common National Base for the Training of Primary Education Teachers, set out in Resolution CNE/CP No. 2 of 20 December 2019, does not ensure the provision of content related to the management of health complications in the school environment^(21)^. Furthermore, Law No. 13.722, which governs the mandatory regular training of teachers and employees, does not specifically address the management of cases of asthma exacerbation, as it refers to first aid in general^(11)^.

The low rate of correct answers to open questions regarding medications was also identified in a recent systematic review on the subject. Of the 13 studies analyzed, six indicated low knowledge of teachers about medications for treating asthma exacerbations^(9)^. A possible hypothesis to explain this finding is that open questions present a higher degree of difficulty when compared to multiple-choice questions, which can be answered randomly and, consequently, with a greater possibility of correct answers.

Quantitative results indicated that teachers had difficulties in answering the question about ways to prevent asthma exacerbation during physical activities; however, the practice of physical exercises was indicated as the main “trigger” when reporting their experiences. A qualitative approach study carried out with 16 physical education teachers pointed out that these are usually the first to assist the child with asthma exacerbation induced by physical exercise, but the majority reported not knowing how to manage the crisis and claimed to have the support of the school nurse in these cases. The physical educators also brought up the challenges related to the facilities of the sports courts, the extreme weather (cold or hot) and the schedule of physical education classes as factors that potentiate asthma attacks during classes^(22)^.

In the present study, teachers with less time in the occupation had higher knowledge scores. However, no studies were found in the literature that could support this finding. In general, the literature indicates that the length of professional experience exerts a positive influence on teachers’ knowledge levels. In this perspective, an international study that sought to evaluate the knowledge and attitudes of teachers regarding diabetes in children found in its results that teachers over 45 years old had greater knowledge about the disease, attributing this better performance to the time of professional experience^(23)^. The use of a technological resource for remote application of the NAQK can be an explanation for this finding, mainly because it required teachers to master the tool, a skill that generally constitutes a challenge for older people.

To reassure the child at the time of the attack, some teachers mentioned the adoption of breathing and relaxation techniques. Such actions corroborate the intervention techniques recommended by the *Global Initiative for Asthma* ^(24)^. According to this document, breathing exercises can be a useful complement to alleviate asthma symptoms and promote a better quality of life for asthma patients. In addition, the document mentions that relaxation helps to reduce stress, being useful in cases of attacks. Moreover, when available in the child’s bag, some teachers immediately administered the medication following another GINA recommendation, which encourages the timely administration of medication during exacerbation of asthma symptoms.

According to multivariate linear regression, teachers who had higher knowledge scores had been diagnosed with asthma. In the same direction, the testimonies pointed out that familiarization with the disease favored safety and tranquility to support the child in crisis. A similar result was evidenced in a Nigerian study where teachers with a personal history of asthma or a family history of asthma performed better^(6)^. Previous experiences with asthmatic children in the family were also listed as a factor associated with better levels of knowledge among Spanish teachers^(17)^.

As most teachers did not have personal or family experiences, the feeling of insecurity and fear prevailed in the testimonies. This finding is in line with the results of a study that analyzed the self-confidence of teachers to handle a situation of health complications in the school environment and concluded that they feel little confident^(25)^. According to some testimonials from teachers, communication is flawed, resulting in teachers not knowing about the child’s asthma diagnosis, increasing fear and insecurity in the face of the disease’s worsening condition. Similar results were found in studies that made up a systematic review on the subject, with the conclusions reinforcing the importance of effective communication between school, family, and health professionals^(9)^.

A Brazilian study dealing with teachers’ knowledge about diabetes mellitus and its management in the school environment, another chronic condition that affects schoolchildren, emphasizes the importance of effective implementation of the PSE to provide adequate integration among education and health professionals and the family^(26)^. In addition to the context of chronic conditions, the literature corroborates by defending the prerogatives of the PSE and emphasizing that nursing care must reach other scenarios beyond those naturally recognized as of care or professional practice, with emphasis on actions related to health in schools^(27)^. In this regard, it is up to the nurse who is part of the primary care health team to strengthen partnerships with the schools in their territory to maximize the full school performance of children with asthma based on actions ruled by health promotion.

Although the results of this study are supported by the literature, it is worth pointing out some limitations. The main one refers to data collection carried out exclusively remotely, which hindered the exchange between the research participant and the researcher, especially in the qualitative stage. Another limiting aspect concerns the researcher’s lack of control over possible searches for information while filling out the NAKQ. Finally, the eligibility of teachers with more than three months of professional experience to participate in the research may have had a negative impact on the number of teachers who claimed to have experienced a situation of asthma exacerbation at school, since teachers hired during the pandemic period mostly worked in remote teaching. Even so, these limitations do not revoke the results of the present research, but indicate the need for future studies.

Finally, this investigation contributes to the advancement of knowledge in nursing due to its uniqueness, by integrating quantitative and qualitative results to explore the teachers’ knowledge and experience with regard to asthma. Moreover, the findings of the present study have the potential to support the planning of health education actions. It is recommended that nurses working in primary care invest in training actions in schools to strengthen the intersectoral articulation between health and education and, consequently, promote qualified assistance to children with asthma in the school environment.

## CONCLUSION

In general, the study found unsatisfactory knowledge about asthma among most teachers. However, those with less professional experience and diagnosed with asthma performed better. The testimonies about the experiences reinforced the quantitative findings, especially when revealing the feelings of tranquility and safety to handle such a situation among teachers who also have asthma.
